# Influencing factors of vascular endothelial function in patients with non-obstructive coronary atherosclerosis: a 1-year observational study

**DOI:** 10.1186/s12872-020-01326-2

**Published:** 2020-01-30

**Authors:** Yin-Ping Li, Zhen-Xing Fan, Jing Gao, Xi-Peng Sun, Guo-Hua Zhu, Ying-Hua Zhang, Jin Si, Xue-Bing Zuo, Zhi Liu, Qi Hua, Jing Li

**Affiliations:** 1 Department of Nephrology, Xuanwu Hospital, Capital Medical University, National Clinical Research Center for Geriatric Diseases, Beijing, 100053 China; 2 Department of Cardiology, Xuanwu Hospital, Capital Medical University, National Clinical Research Center for Geriatric Diseases, Beijing, 100053 China

**Keywords:** Non-obstructive coronary atherosclerosis, Endothelial dysfunction, Flow-mediated dilatation, Endothelial progenitor cells

## Abstract

**Background:**

Endothelial dysfunction may play a key role in non-obstructive coronary artery atherosclerosis. Our study aimed to evaluate the vascular endothelial function and its influencing factors in patients with non-obstructive coronary artery atherosclerosis.

**Methods:**

A total of 131 consecutive patients with non-obstructive coronary artery atherosclerosis were enrolled. Flow-mediated dilatation (FMD) was measured at baseline and 1-year follow-up. Endothelial progenitor cells (EPCs) were counted by staining the fasting venous blood with antibodies against CD34 and vascular endothelial growth factor receptor 2.

**Results:**

Systolic blood pressure, pulse pressure and the levels of HbA1c in participants with baseline FMD < 6% (*n* = 65) were significantly higher than those with baseline FMD ≥ 6% (*n* = 66). Baseline FMD was negatively associated with EPC counts (r = − 0.199, *P* < 0.05) and systolic blood pressure (r = − 0.315, *P* < 0.01). The 1-year FMD was significantly increased compared to the baseline FMD [(9.31 ± 5.62) % vs (7.31 ± 5.26) %, *P* < 0.001]. Independent predictors of FMD improvement included elevated EPC counts (OR = 1.104, 95% CI: 1.047–1.165, *P* < 0.001) and decreased levels of serum creatinine (OR = 0.915, 95% CI: 0.843–0.993, *P* = 0.034).

**Conclusions:**

Family history of premature cardiovascular diseases, hypertension, elevated systolic pressure, and HbA1c > 6.5% are independent risk factors for endothelial dysfunction in non-obstructive atherosclerotic patients. Elevated peripheral blood EPC counts and decreased levels of serum creatinine are independent predictors of endothelial function improvement.

## Introduction

Non-obstructive coronary artery atherosclerosis is characterized by coronary artery stenosis less than 50% [1–3]. Vascular endothelial dysfunction, plaque rupture and thrombosis may be the main pathologic mechanism of acute or chronic myocardial ischemia [4, 5]. The vascular endothelium is a multifunctional organ that maintains vascular homeostasis, regulates cell proliferation and angiogenesis and preserves a non-thrombogenic blood-tissue interface. Endothelium dysfunction may play a key role in non-obstructive coronary artery atherosclerosis [6, 7]. Previous studies suggested that impaired endothelial function may be reversed by medicine treatment and lifestyle changes [8, 9].

Endothelial function can be directly evaluated by measuring the changes of arterial diameter in response to vasoactive drugs like nitric oxide that is directly injected into the coronary artery or the brachial artery [10–12]. However, this method is poorly accepted due to its invasiveness and high cost. On the contrary, flow-mediated dilatation (FMD) [13–15] and vascular reactive hyperemia index are noninvasive methods for evaluating endothelial function. They both use the principle of endothelium-dependent vessel diastolic function [16].

It has been shown that high-sensitivity C-reactive protein levels and neutrophil-to-lymphocyte ratio are significantly associated with vascular endothelial dysfunction [17, 18]. Endothelial cells can be regenerated by bone marrow-derived circulating endothelial progenitor cells (EPCs), which accelerates endothelialization and prevents atherosclerosis [19, 20]. Elevated EPC counts are thought to represent the ability of endothelial repair and atherosclerosis inhibition [21].

Our study aimed to investigate the influencing factors of vascular endothelial function in patients with non-obstructive coronary artery atherosclerosis.

## Methods

### Participants

The prospective observation study included 131 consecutive patients with non-obstructive coronary artery atherosclerosis with atypical symptoms and/or non-specific electrocardiogram changes at the Xuanwu Hospital of Capital Medical University. Non-obstructive coronary atherosclerosis was diagnosed using angiography as the absence of obstructive coronary artery disease, i.e. no coronary artery stenosis ≥50% in any coronary artery. This includes patients with normal coronary arteries (no stenosis to stenosis < 30%) or mild coronary atheromatosis (stenosis of 30 to 50%) [PMID: 28158518]. The study was carried out from August 2013 to August 2015. All patients underwent coronary angiography or coronary computed tomographic angiography and had been conformed with coronary stenosis < 50%.

Patients with the following conditions were excluded: (1) coronary artery stenosis > 50% shown by imaging, or previous history of coronary artery interventional therapy or coronary artery bypass graft; (2) previous positive treadmill test, or transient elevation of ST-segment; (3) previous imaging suggesting myocardial ischemia; (4) previous tests showing levels of troponin I, troponin T, or creatine kinase-MB exceeding the upper limits of normal ranges; (5) patients with symptomatic heart failure, atrial fibrillation, cardiomyopathy or valvular diseases; (6) history of aortic dissection aneurysm, stroke or symptomatic peripheral artery diseases; (7) surgery, trauma or infection within 30 days; (8) renal or liver dysfunction; (9) secondary hypertension, hypertension emergency, or diabetes emergency; (10) rheumatic diseases, cancers, thyroid dysfunction, severe anemia, or use of glucocorticoid; (11) the informed consents were not signed.

### Data collection and follow-up

Patient general information such as gender, age, smoking, height and weight were collected. The family history of premature cardiovascular disease was defined as having a first-degree male relative aged < 55 years and/or a first-degree female relative aged < 65 years.

Blood pressure was measured using a mercury sphygmomanometer for three times with intervals of 2 min [22]. The mean blood pressure and the pulse pressure were calculated. Fasting blood was drawn from the median cubital vein in the morning. Blood routine was performed by a hematology analyzer (Sysmex XE-2100). Serum levels of glycated hemoglobin (HbA1c), homocysteine and high-sensitivity C-reactive protein were measured by a biochemical analyzer (HITACHI 7600). To exclude myocardial injury/infarction, levels of cardiac troponin I were measured by a triage quantitative myocardial infarction/heart failure diagnostic device (Biosite, USA).

Patients were followed 1 year ±30 days later from the time of enrollment. The following events during the year were registered: myocardial infarction, percutaneous coronary intervention, coronary artery bypass grafting, heart failure, angina, hospitalization related to cardiovascular diseases, stroke (ischemic and/or hemorrhagic), transient ischemia attack, cardiac death and all-cause death.

### Evaluation of *vascular endothelial function*

Vascular endothelial function was evaluated using the flow-mediated dilatation (FMD) on the day after admission and on the morning of follow-up visit [23]. Participants were required to fasted for 8 h and to avoided exercising, smoking, drinking, coffee, tea, and high-fat food (at least 12 h). Medicines that contain vasoactive agents were stopped for at least 24 h. The evaluation was performed by one investigator blinded to the study design in a quiet room after rest for 20 min. Color Doppler ultrasonography of the brachial artery was performed using a L12–3 transducer (10–13 MHz, Philips IE33). Electrocardiogram was recorded synchronously. FMD was calculated using the following formula: FMD = (D1 - D0) / D0 × 100%. D1 was the inner diameter of the brachial artery at the end of diastole. D0 was the basal inner diameter of the brachial artery. FMD < 6% was considered abnormal [24]. ΔFMD was calculated using the formula: ΔFMD = (1-year FMD - baseline FMD) / baseline FMD × 100%.

### EPC counting

EPCs in the whole blood were identified and counted by detecting the expression of CD34 and vascular endothelial growth factor receptor 2 (VEGFR2) using flow cytometry (BD Biosciences, USA) [25–27]. The data were processed by the BD FACS Diva software (BD Biosciences, USA).

### Statistical analysis

Data normality was examined using the Kolmogorov-Smirnov test. Quantitative variables with a normal distribution were presented as means ± standard deviations. Variables with a skewed distribution were presented as medians (interquartile ranges). Categorical variables were shown as numbers and percentage values.

One-way analysis of variance test or Kruskal-Wallis test was used to compare the continuous variables. Chi-square test or Fisher’s exact test was used for comparing the categorical variables. Spearman or Pearson correlation coefficient was used to represent the relationships between the variables.

Multivariate logistic regression was used to analyze the influencing factors of FMD. The dependent variable was FMD, and the independent variables were age > 65, male gender, smoking, hypertension, family history of premature cardiovascular diseases, HbA1c ≥ 6.5%, total cholesterol, low-density lipoprotein cholesterol, systolic blood pressure, pulse pressure and EPC count.

Differences were considered statistically significant if the two-sided *P* < 0.05. All analyses were performed with SPSS statistical software version 19.0 (SPSS, Chicago, IL).

## Results

Our study included 131 consecutive patients with non-obstructive coronary artery atherosclerosis (Tables 1). The male to female ratio was 1:1.11. FMD was (7.31 ± 5.26) % at baseline. FMD ≥ 6% was considered to suggest a normal endothelial function. The general information and laboratory results were compared between patients with FMD < 6% and those with FMD ≥ 6% (Table 2). No significant difference in inflammatory biomarkers and CD34 + VEGFR2+ EPC counts was found between patients with FMD < 6% and those with FMD ≥ 6% (Table 2).

**Table 1:** General information of the participants

	Participants (*n* = 131)
Age, year	60.15 ± 11.17
Male, n (%)	62 (47.3)
Body mass index, kg/m^2^	25.64 ± 3.52
Heart rate, beats/min	71.87 ± 10.49
Systolic blood pressure, mmHg	134.54 ± 16.72
Diastolic blood pressure, mmHg	75.32 ± 10.55
Pulse pressure, mmHg	58.62 ± 16.28
Homocysteine, μmol/L	14.01 ± 8.27
Glucose, mmol/L	5.54 ± 1.49
HbA1c, %	6.16 ± 0.85
Serum creatinine, μmol/L	66.59 ± 15.58
Uric acid, μmol/L	334.27 ± 93.42
Total cholesterol, mmol/L	4.19 ± 0.84
Triglyceride, mmol/L	1.77 ± 1.00
High-density lipoprotein cholesterol, mmol/L	1.33 ± 0.34
Low-density lipoprotein cholesterol, mmol/L	2.39 ± 0.68
Ejection fraction, %	65.69 ± 7.14
Flow-mediated dilatation, %	7.311 ± 5.259
Hypertension	68 (51.9)
Diabetes mellitus	30 (23.3)
Smoking	28 (21.4)
Family history of premature cardiovascular disease	27 (20.6)
Antiplatelet therapy	35 (26.7)
β-blocker	28 (21.4)
Calcium antagonist	35 (26.7)
Angiotensin-converting-enzyme inhibitors / angiotensin II receptor blockers	35 (26.7)
Diuretics	10 (7.6)
Statins	28 (21.4)
Hypoglycemic agents	22 (16.8)

**Table 2:** Comparison between patients with FMD < 6% and those with FMD ≥ 6%

	FMD < 6% (*n* = 65)	FMD ≥ 6% (*n* = 66)	*P*-value
FMD, %	3.11 ± 1.69	11.45 ± 4.17	< 0.001
Age, year	60.74 ± 11.41	59.56 ± 10.99	0.548
Male, n (%)	30 (46.2)	32 (48.5)	0.771
Body mass index, kg/m^2^	25.82 ± 3.86	25.46 ± 3.16	0.584
Smoking, n (%)	13 (20.3)	15 (23.1)	0.689
Diabetes mellitus, n (%)	19 (29.7)	11 (16.9)	0.121
Family history of premature cardiovascular disease, n (%)	13 (20.0)	14 (21.2)	0.839
Hypertension, n (%)	33 (50.8)	35 (53.0)	0.778
Statins, n (%)	10 (15.4)	18 (27.3)	0.106
β-blocker, n (%)	16 (24.6)	12 (18.8)	0.419
Calcium channel blockers, n (%)	16 (24.6)	19 (28.8)	0.589
Angiotensin-converting enzyme inhibitors / angiotensin II receptor blockers, n (%)	19 (29.2)	16 (24.2)	0.519
Heart rate, beats/min	70.98 ± 11.42	72.73 ± 9.52	0.351
Systolic blood pressure, mmHg	138.38 ± 16.41	130.69 ± 16.25	0.008
Diastolic blood pressure, mmHg	74.75 ± 10.72	75.88 ± 10.43	0.546
Pulse pressure, mmHg	63.75 ± 16.29	54.89 ± 14.75	0.001
Serum creatinine, μmol/L	65.64 ± 17.08	67.53 ± 14.02	0.497
Uric acid, mmol/L	338.73 ± 109.08	329.95 ± 75.89	0.600
Glucose, mmol/L	5.68 ± 1.60	5.40 ± 1.37	0.290
HbA1c, %	6.49 ± 0.94	5.79 ± 0.53	< 0.001
Total cholesterol, mmol/L	1.79 ± 1.19	1.75 ± 0.78	0.822
Triglyceride, mmol/L	4.04 ± 0.76	4.33 ± 0.89	0.058
High-density lipoprotein cholesterol, mmol/L	1.30 ± 0.36	1.35 ± 0.32	0.493
Low-density lipoprotein cholesterol, mmol/L	2.26 ± 0.62	2.51 ± 0.70	0.035
Homocysteine, μmol/L	15.11 ± 12.04	13.22 ± 3.92	0.439
Ejection fraction, %	66.90 ± 6.58	64.46 ± 7.52	0.093
White blood cell, ×10^9^/L	6.27 ± 1.71	6.87 ± 1.94	0.068
Neutrophil, ×10^9^/L	3.55 ± 1.49	3.97 ± 1.68	0.140
Neutrophil-to-lymphocyte ratio	2.08 ± 1.25	1.97 ± 1.12	0.618
Red blood cell distribution width, %	12.73 ± 2.76	12.93 ± 0.73	0.818
High-sensitivity C-reactive protein, mg/L	3.37 ± 4.13	7.87 ± 14.31	0.160
Endothelial progenitor cells per 10^6^ cells	52.00 ± 22.13	46.59 ± 24.78	0.254

*FMD* flow-mediated dilatation

The mean EPC count was 43 cells per 10^6^ cells. Heart rate, systolic blood pressure and pulse pressure in patients with EPC counts < 43 cells per 10^6^ cells were significantly lower than that in those with EPC counts ≥43 cells per 10^6^ cells. FMD in patients with lower EPC counts was significantly higher than that in those with higher EPC counts (Table 3). There was no significant difference in inflammatory factors between patients with lower EPC counts and those with higher EPC counts (Table 3). Pearson correlation analysis showed that EPC count was negatively associated with FMD (r = − 0.199, *P* < 0.05), and that FMD was negatively associated with systolic blood pressure (r = − 0.315, *P* < 0.01). However, no significant correlation was noticed between EPC count and systolic blood pressure/heart rate/white blood cell count, FMD and heart rate/white blood cell count, and systolic blood pressure and heart rate/white blood cell count.

**Table 3:** Comparison between patient with lower EPC counts and those with higher EPC counts

	EPC counts < 43 cells per 10^6^ cells (*n* = 65)	EPC counts ≥43 cells per 10^6^ cells (*n* = 66)	*P*-value
Flow-mediated dilatation, %	9.16 ± 5.86	6.47 ± 4.33	0.009
Age, year	59.38 ± 11.04	59.98 ± 11.02	0.784
Male, n (%)	29 (44.6)	33 (50.0)	0.537
Body mass index, kg/m^2^	25.47 ± 3.62	25.60 ± 3.40	0.858
Smoking, n (%)	16 (24.6)	12 (18.2)	0.369
Diabetes mellitus, n (%)	16 (24.6)	14 (21.2)	0.643
Family history of premature cardiovascular disease, n (%)	13 (20.0)	14 (21.2)	0.864
Hypertension, n (%)	33 (50.8)	35 (53.0)	0.796
Heart rate, beats/min	69.04 ± 9.11	74.14 ± 10.64	0.012
Systolic blood pressure, mmHg	130.16 ± 16.55	137.29 ± 15.14	0.026
Diastolic blood pressure, mmHg	75.14 ± 8.53	74.31 ± 10.81	0.669
Pulse pressure, mmHg	54.41 ± 15.08	61.92 ± 16.53	0.020
Serum creatinine, μmol/L	66.76 ± 13.97	65.23 ± 17.01	0.627
Uric acid, mmol/L	313.33 ± 86.46	337.66 ± 95.91	0.188
Glucose, mmol/L	5.40 ± 1.12	5.54 ± 1.67	0.608
HbA1c, %	6.00 ± 0.55	6.29 ± 1.11	0.148
Total cholesterol, mmol/L	1.68 ± 0.69	1.70 ± 0.85	0.882
Triglyceride, mmol/L	4.20 ± 0.90	4.25 ± 0.87	0.805
High-density lipoprotein cholesterol, mmol/L	1.31 ± 0.35	1.35 ± 0.34	0.589
Low-density lipoprotein cholesterol, mmol/L	2.44 ± 0.72	2.44 ± 0.67	0.952
Homocysteine, μmol/L	13.24 ± 4.01	13.32 ± 3.99	0.439
Ejection fraction, %	65.54 ± 6.27	64.82 ± 7.55	0.957
White blood cell, ×10^9^/L	6.13 ± 2.34	6.75 ± 2.17	0.118
Neutrophil, × 10^9^/L	3.51 ± 1.58	3.88 ± 1.86	0.222
Neutrophil-to-lymphocyte ratio	2.08 ± 1.25	1.97 ± 1.12	0.057
Red blood cell distribution width, %	12.78 ± 0.76	12.96 ± 0.79	0.270
High-sensitivity C-reactive protein, mg/L	5.24 ± 11.52	6.33 ± 11.62	0.802

*EPC* endothelial progenitor cell

Multivariate logistic regression showed that hypertension (odds ratio [OR] = 24.335, 95% confidence interval [CI]: 2.467–240.048), family history of premature cardiovascular (OR = 0.068, 95% CI 0.006–0.720), HbA1c ≥ 6.5% (OR = 0.059, 95% CI 0.007–0.485) and elevated systolic blood pressure (OR = 0.902, 95% CI: 0.821–0.990) were independently related to FMD decline at 1-year follow-up (Table 4).

**Table 4:** Multivariate logistic regression analysis of influencing factors of FMD decline at 1-year follow-up

	B	S.E.	Wald	P	Exp (B)	95% CI
Age ≥ 65 years	−1.369	1.198	1.306	0.253	0.254	0.024–2.661
Male gender	−0.619	0.844	0.539	0.463	0.538	0.103–2.815
Hypertension	3.192	1.168	7.470	0.006	24.335	2.467–240.048
Family history of premature cardiovascular diseases	−2.685	1.202	4.987	0.026	0.068	0.006–0.720
Smoking	0.412	1.179	0.122	0.727	1.510	0.150–15.231
HbA1c ≥ 6.5%	−2.829	1.075	6.934	0.008	0.059	0.007–0.485
Total cholesterol	1.078	1.190	0.821	0.365	2.939	0.285–30.277
Low-density lipoprotein cholesterol	−0.582	1.566	0.138	0.710	0.559	0.026–12.030
Systolic blood pressure	−0.103	0.048	4.668	0.031	0.902	0.821–0.990
Pulse pressure	−0.029	0.040	0.526	0.468	0.971	0.898–1.051
Endothelial progenitor cells	0.001	0.014	0.006	0.941	1.001	0.975–1.028
Constant	15.174	5.853	6.722	0.010	3,889,333.926	

*FMD* flow-mediated dilatation

Five participants were lost to follow-up (3.82%). The 1-year FMD was significantly improved from the baseline [(9.31 ± 5.62) % vs (7.31 + 5.26) %, *P* < 0.001). The use of antiplatelet therapy, angiotensin-converting-enzyme inhibitor / angiotensin II receptor blockers, β-blockers and statins were significantly higher at 1-year follow-up than that at baseline (Table 5).

**Table 5:** Medications at baseline and 1-year follow-up [n (%)]

Medications	Baseline (*n* = 131)	1-year follow-up (*n* = 126)	*P*-value
Antiplatelet therapy	35 (26.7)	85 (67.5)	< 0.001
β-blocker	28 (21.4)	68 (54.0)	< 0.001
Calcium channel blockers	35 (26.7)	28 (22.2)	0.312
ACE-I/ARB	35 (26.7)	56 (44.4)	0.003
Diuretics	10 (7.6)	10 (7.9)	1
Statins	28 (21.4)	86 (68.3)	< 0.001
Hypoglycemic agents	22 (16.8)	29 (23.0)	0.215

*ACE-I/ARB* angiotensin-converting enzyme inhibitors / angiotensin II receptor blockers

Participants with ΔFMD ≥10% had significantly higher proportions of hypertension, elevated systolic blood pressure, elevated pulse pressure and lower baseline FMD than those ΔFMD < 10%. Participants with ΔFMD < 10% had significantly more patients with diabetes and hypoglycemic therapy (biguanides, sulfonylureas, glinides and alpha-glucosidase inhibitors) than those with ΔFMD ≥10% (Table 6). EPC counts in participants with ΔFMD ≥10% was significantly higher than those with ΔFMD < 10% (59.14 ± 24.36 per 10^6^ cells vs 36.11 ± 15.16 per 10^6^ cells) at baseline (Table 6).

**Table 6:** Comparison between participants with ΔFMD < 10% and those with ΔFMD ≥10%

	ΔFMD < 10% (*n* = 55)	ΔFMD ≥10% (*n* = 71)	*P*-value
Baseline FMD, %	9.92 ± 5.48	6.11 ± 4.51	< 0.001
1-year FMD, %	9.35 ± 5.71	9.28 ± 5.61	0.951
Age, years	58.62 ± 10.74	60.51 ± 11.19	0.387
Male, n (%)	27 (49.1)	35 (49.3)	0.944
Body mass index, kg/m^2^	25.60 ± 3.69	25.50 ± 3.37	0.891
Smoking, n (%)	14 (25.5)	14 (19.7)	0.803
Diabetes mellitus, n (%)	19 (34.5)	11 (15.5)	0.017
Family history of premature cardiovascular diseases, n (%)	14 (25.5)	13 (18.3)	0.644
Hypertension, n (%)	23 (41.8)	45 (63.4)	0.012
Heart rate, beats/min	71.66 ± 10.40	71.58 ± 10.12	0.970
Systolic blood pressure, mmHg	129.14 ± 16.95	137.46 ± 14.66	0.010
Diastolic blood pressure, mmHg	75.09 ± 8.66	74.64 ± 10.87	0.733
Pulse pressure, mmHg	54.05 ± 14.60	63.04 ± 16.04	0.005
Serum creatinine, μmol/L	69.02 ± 14.41	63.55 ± 16.07	0.081
Uric acid, mmol/L	330.23 ± 80.96	321.93 ± 100.05	0.657
Glucose, mmol/L	5.41 ± 1.63	5.52 ± 1.21	0.715
HbA1c, %	6.06 ± 1.07	6.21 ± 0.70	0.468
Total cholesterol, mmol/L	1.72 ± 0.69	1.68 ± 0.84	0.795
Triglyceride, mmol/L	4.24 ± 0.88	4.21 ± 0.89	0.884
High-density lipoprotein cholesterol, mmol/L	1.27 ± 0.31	1.38 ± 0.37	0.116
Low-density lipoprotein cholesterol, mmol/L	2.50 ± 0.74	2.39 ± 0.65	0.448
Homocysteine, μmol/L	12.28 ± 2.89	14.53 ± 5.69	0.132
Ejection fraction, %	64.26 ± 6.52	65.95 ± 7.24	0.291
Medications	ΔFMD < 10% (n = 55)	ΔFMD ≥10% (n = 71)	P-value
Antiplatelet therapy	37 (67.2)	48 (67.6)	0.879
ACE-I/ARB	21 (38.2)	35 (49.3)	0.193
β-blockers	27 (49.1)	41 (57.7)	0.291
Calcium channel blockers	11 (20.0)	17 (23.9)	0.549
Diuretics	4 (7.3)	6 (8.5)	0.777
Statins	37 (67.3)	49 (69.0)	0.462
Hypoglycemic agents	18 (32.7)	11 (15.5)	0.017
White blood cell, ×10^9^/L	6.35 ± 1.42	6.87 ± 1.94	0.542
Neutrophil, ×10^9^/L	3.53 ± 1.37	3.83 ± 1.70	0.352
Lymphocytes, ×10^9^/L	2.23 ± 0.90	2.02 ± 0.62	0.169
Neutrophil-to-lymphocyte ratio	1.92 ± 1.35	2.00 ± 0.92	0.736
Red blood cell distribution width, %	12.76 ± 0.63	12.96 ± 0.87	0.191
High-sensitivity C-reactive protein, mg/L	5.65 ± 11.42	5.95 ± 11.74	0.944
Endothelial progenitor cells per 10^6^ cells	36.11 ± 15.16	59.14 ± 24.36	< 0.001

*FMD* flow-mediated dilatation; *ACE-I/ARB, ACE-I/ARB* angiotensin-converting-enzyme inhibitors / angiotensin II receptor blockers

Multivariate logistic regression analysis showed that elevated EPC counts (OR = 1.104, 95% CI: 1.047–1.165) and decreased levels of serum creatinine (OR = 0.915, 95% CI: 0.843–0.993) were independently associated with FMD improvement at 1-year follow-up (Table 7).

**Table 7:** Multivariate logistic regression analysis of influencing factors of FMD improvement at 1-year follow-up

	B	S.E.	Wald	P	Exp (B)	95% CI
Male gender	−1.126	1.163	0.937	0.333	0.324	0.033–3.171
Age ≥ 65 years	1.798	0.938	3.673	0.055	6.038	0.960–37.974
Family history of premature cardiovascular diseases	−0.691	0.849	0.661	0.416	0.501	0.095–2.648
Smoking	−0.176	0.975	0.033	0.856	0.838	0.124–5.666
Systolic blood pressure	0.041	0.025	2.793	0.095	1.042	0.993–1.094
Low-density lipoprotein cholesterol	−0.471	0.687	0.470	0.493	0.625	0.163–2.400
Serum creatine	−0.089	0.042	4.479	0.034	0.915	0.843–0.993
HbA1c ≥ 6.5%	−0.686	0.518	1.754	0.185	0.503	0.182–1.390
Endothelial progenitor cells	0.099	0.027	13.295	0.000	1.104	1.047–1.165
Constant	3.208	5.772	0.309	0.578	24.725	

*FMD* flow-mediated dilatation

## Discussion

Increased blood flow-associated shear stress in hypertensive patients can significantly affect endothelial permeability [28, 29]. Our study found that systolic blood pressure and pulse pressure were significantly higher in the participants with FMD < 6% than those with FMD ≥ 6%. We also found that hypertension, systolic blood pressure and pulse pressure were independent risk factors in predicting endothelial dysfunction. It has been suggested that oxidative stress and endothelial dysfunction are associated with impaired vasodilatory capacity, which leads to hypertension [PMID: 28035582, 25,136,585, 27,203,578]. In addition, endothelial dysfunction is also associated with increased pulse pressure and hypertension in type 1 diabetes [PMID: 29101422].

Our study included 30 participants with diabetes and found elevated HbA1c levels were an independent influencing factor of endothelial dysfunction, suggesting diabetes may be associated with endothelial dysfunction. Hyperglycemia in diabetes is associated with inflammation and oxidative stress, which can result in endothelial dysfunction [PMID: 26781070, 30,274,207].

It has been shown that the phenotypic EPCs are independently associated with the severity of coronary artery lesion and carotid intima-media thickness and can be used as an independent predictor of cardiovascular outcomes [30, 31]. Our study found that the CD34 + VEGFR2+ EPC count was associated with the baseline FMD. Heart rate, systolic blood pressure and pulse pressure in participants with higher EPC counts were significantly higher than that in those with lower EPC counts. These results suggest that elevated systolic blood pressure and pulse pressure were more likely to be associated with differentiation and release of bone marrow-derived EPCs into the blood in comparison with to other risk factors of endothelial dysfunction. However, multivariate logistic regression analysis did not find independent association between EPC counts and baseline FMD.

A previous study found that high-sensitivity C-reactive protein was an independent risk factor for coronary heart disease and its level was significantly associated with the risk of future cardiovascular events, such as sudden death, acute myocardial infarction, and peripheral vascular disease [32, 33]. Another study showed that neutrophil-to-lymphocyte ratio was significantly associated with urinary albumin-to-creatinine ratio in asymptomatic stable coronary heart disease populations and was an independent predictor of systemic endothelial dysfunction [34, 35]. Neutrophil-to-lymphocyte ratio was independently associated to endothelial dysfunction and could predict composite cardiovascular endpoints [36, 37]. However, our study found that high-sensitivity C-reactive protein, white blood cell count and neutrophil-to-lymphocyte ratio were not significantly associated with FMD, suggesting that these inflammatory factors have no definite diagnostic value in low-risk patients with non-obstructive coronary atherosclerosis.

All our participants received intensive blood-pressure control, antiplatelet and statins therapy. No major cardiovascular events occurred during the 1-year follow-up. We found participants with worse baseline endothelial function had greater increase in 1-year FMD. Our study suggests that patients with hypertension, elevated systolic blood pressure and elevated pulse pressure at baseline are more likely to benefit from antihypertensive treatment. It has been shown that aliskiren, a direct renin inhibitor, can improve endothelial function and arterial stiffness when being used as an antihypertensive agent [PMID: 24994608, 24,708,382]. Another study showed that bisoprolol improved endothelial function in patients with hypertension and stable angina [PMID: 23609363]. Further research is needed to illustrate the detailed mechanisms between antihypertensive treatment and endothelial dysfunction.

In our study, participants with diabetes and elevated HbA1c did not show FMD improvements despite the same antihypertensive, antiplatelet and antihyperlipidemic treatments with the non-diabetic participants. We speculate the conventional antidiabetic medications have limited protective effect for vascular endothelial function. Diabetes can induce endothelial dysfunction, leading to increased risks of cardiovascular diseases [PMID: 28044409]. In addition, hyperglycemia is associated with EPCs dysfunction and endothelial dysfunction [PMID: 28718318]. Dapagliflozin is used to treated type 2 diabetes and showed improvements in endothelial function and arterial stiffness [PMID: 29061124]. New antidiabetic drugs, such as dipeptidyl peptidase-4 inhibitors, sodium-glucose cotransporter-2 inhibitors and glucagon-like peptide-1 receptor agonists also showed differentia effect on endothelial function and arterial stiffness [PMID: 30622967].

Chronic kidney disease and cardiovascular disease share similar risk factors. It has been shown that vascular endothelial function and FMD decreased significantly in patients with end-stage renal disease [38, 39]. Similarly, our study found that elevated levels of serum creatinine were associated with continuous endothelial injury. We speculate that major cardiovascular risk factors such as hypertension, diabetes and renal dysfunction can aggravate atherosclerosis partly by impairing the endothelial function. Our findings suggest that vascular endothelial dysfunction induced by hypertension is can be improved with antihypertensive treatment, while that associated with diabetes and renal dysfunction is more difficult to reverse.

Our study has limitations. Our study excluded patients with non-obstructive coronary atherosclerosis who present with symptoms of typical myocardial ischemia and acute coronary syndrome, suggesting worse endothelial dysfunction. This may underestimate the incidence and severity of endothelial dysfunction in patients with early atherosclerosis. At the 1-year follow-up, blood pressure, low-density lipoprotein cholesterol and HbA1c were not included in the analysis, making it difficult to assess the effect of anti-atherosclerosis treatment on vascular endothelial function.

## Conclusion

Family history of premature cardiovascular diseases, hypertension, elevated systolic pressure, and HbA1c > 6.5% are independent risk factors for endothelial dysfunction in non-obstructive atherosclerotic patients. Elevated circulating EPC counts and decreased levels of serum creatinine are independent predictors of endothelial function improvement. Our findings may help to facilitate the risk stratification of patients with mild coronary atherosclerosis, and to explore intervention methods to repair vascular endothelial function.

## Data Availability

The datasets used and/or analyzed during the current study will be available from the corresponding author on reasonable request.

## Abbreviations

EPCs: Endothelial progenitor cells
FMD: Flow-mediated dilatation
VEGFR2: Vascular endothelial growth factor receptor 2
